# Reassessment of Neuroprotective Efficacy of TUDCA in Ischemic Stroke: A Multi-Model In Vivo and In Vitro Study

**DOI:** 10.3390/ijms27198702

**Published:** 2026-09-29

**Authors:** Seyedeh Maryam Mousavi, Sumeyra Nuran Ozmen, Mujeeb Adekunle Adedokun, Gulin Baran, Ahmet Baki Sahin, Lorenz Hirt, Nur Mustafaoglu, Corinne Benakis

**Affiliations:** 1 Stroke Laboratory, Neurology Service, Department of Clinical Neurosciences, Lausanne University Hospital Centre, University of Lausanne, CH-1011 Lausanne, Switzerland; seyedeh-maryam.mousavi@unil.ch (S.M.M.); lorenz.hirt@chuv.ch (L.H.); 2 Department of Fundamental Neurosciences, University of Lausanne, CH-1005 Lausanne, Switzerland; 3 Faculty of Engineering and Natural Sciences, Sabanci University, Tuzla 34956, Istanbul, Türkiye; sumeyra.ozmen@sabanciuniv.edu (S.N.O.); gulin.baran@sabanciuniv.edu (G.B.); ahmet.sahin@sabanciuniv.edu (A.B.S.); 4 Institute for Stroke and Dementia Research (ISD), LMU University Hospital, LMU Medizin, LMU Munich, 81377 Munich, Germany; mujeeb.adedokun@med.uni-muenchen.de; 5 Sabanci University Nanotechnology Research and Application Center (SUNUM), Tuzla 34956, Istanbul, Türkiye; 6 Centre of Excellence for Functional Surfaces and Interfaces for Nano Diagnostics (EFSUN), Sabanci University, Tuzla 34956, Istanbul, Türkiye

**Keywords:** ischemic stroke, TUDCA, blood-brain barrier, MCAO, organ-on-chip

## Abstract

Ischemic stroke accounts for 87% of all strokes and remains a leading cause of mortality and morbidity worldwide, yet therapeutic options remain limited. Emerging evidence on the gut-brain axis and microbial metabolites as modulators of stroke outcome has positioned tauroursodeoxycholic acid (TUDCA), a taurine-conjugated secondary bile acid, as a candidate neuroprotective agent. Here, we evaluated the effects of intravenous TUDCA (100 mg/kg) in male C57BL/6 mice subjected to transient middle cerebral artery occlusion (tMCAO) or permanent distal middle cerebral artery occlusion (dMCAO), administered either one hour before or after stroke induction. Infarct size, neurological score, and IgG extravasation served as primary outcome measures, and blood-brain barrier (BBB) integrity was further assessed using a human iPSC-derived BBB organ-on-chip system. TUDCA did not significantly reduce infarct volume, improve neurological outcome, or attenuate BBB leakage in either in vivo model, nor did it preserve barrier permeability or ZO-1 expression in vitro under normoxic or hypoxic conditions. These findings indicate that, under the conditions tested, TUDCA does not influence the development of ischemic brain injury or features of BBB integrity, and that further optimization of dosing and administration strategies is required to fully evaluate its therapeutic potential in ischemic stroke.

## 1. Introduction

Bile acids, long considered simply detergent molecules facilitating lipid metabolism, are now recognized as vital regulators of various biological processes [1]. Among them, tauroursodeoxycholic acid (TUDCA), a taurine-conjugated form of ursodeoxycholic acid [2], has received considerable attention due to its ability to cross the blood-brain barrier (BBB), mainly owing to its hydrophilic nature as well as its low toxicity profile [3,4]. Moreover, TUDCA has been demonstrated to exert therapeutic effects in several neurological disorders, including Alzheimer’s disease [5], Parkinson’s disease [6], and Huntington’s disease [7], and in different models of cerebral injury [8,9], mainly by modulating endoplasmic reticulum (ER) stress, mitochondrial dysfunction, and apoptosis [10].

Despite the sometimes very efficient therapies aimed at restoring cerebral blood flow, ischemic stroke is one of the leading causes of death and long-term disability worldwide, yet effective neuroprotective therapies remain unavailable [11]. A critical mediator of post-ischemic secondary injury is the biphasic disruption of the BBB [12]. An early phase of barrier opening occurs within hours of ischemia onset, driven primarily by oxidative stress and the activation of matrix metalloproteinases (MMP-9, MMP-3), which degrade tight junction proteins (claudin-5, occludin, ZO-1) and basal lamina components [13,14]. Later, a more pronounced phase of barrier disruption follows at approximately 24–72 h, coinciding with neuroinflammatory amplification and further tight junction degradation, which is later accompanied by immunoglobulin G (IgG) extravasation and increased infarct expansion [15,16,17]. This temporal profile defines a critical therapeutic window in which interventions that maintain BBB integrity have the potential to limit secondary injury. On the other hand, the gut-brain axis has emerged as a key systemic modulator of stroke outcome progression. Cerebral ischemia triggers intestinal dysbiosis and barrier dysregulation, thereby priming gut-resident immune populations that traffic to the ischemic brain and exacerbate neuroinflammation [18]. In return, microbiota-derived metabolites regulate BBB integrity and microglial activation, identifying the gut-brain axis as a potential therapeutic target [19].

TUDCA, due to its anti-apoptotic and anti-ER stress characteristics, can represent a promising target for modulating stroke-induced impairments [8,9]. In rat models of ischemic stroke, TUDCA has been injected intravenously at doses ranging from 64 to 400 mg/kg, with lower doses (64–180 mg/kg) exerting partial (30–40%) lesion reduction and administration of the highest dose (400 mg/kg) resulting in a decrease of around 50% when given during or within 1 h after reperfusion [8]. Similarly, in a rat model of acute haemorrhagic stroke, 10–200 mg/kg of TUDCA administered 1 h before or up to 6 h after induction of injury has similarly shown neuroprotection [9]. However, whether these findings are reproducible in mice has not been established. TUDCA has also been shown to restore gut epithelial barrier function [20]. Despite these promising mechanisms, evidence supporting a protective role of TUDCA across different models of injury and disease remains limited and inconsistent [21,22,23]. In particular, its effects on BBB integrity have not been systematically investigated, and it remains unclear whether its potential benefits are consistent across different models of cerebral ischemia.

To address this, we have implemented transient middle cerebral artery occlusion (tMCAO) and distal MCAO (dMCAO) in this study to represent distinct anatomical and pathophysiological aspects of ischemic stroke. While tMCAO is characterized by the temporary occlusion of the MCA, which represents ischemia-reperfusion injury that mimics the clinical reperfusion and recanalization treatments [24], dMCAO induces sustained cerebral ischemia without restoration of blood flow, thereby primarily modelling perpetual vessel occlusion and injury driven by prolonged energy deprivation and excitotoxicity [25]. Together, these models allow us to study both ischemic and reperfusion-associated pathologies in mice. In these in vivo models, pre-ischemic TUDCA administration was designed to model a prophylactic scenario relevant to patients at known ischemic risk (e.g., cardiac or carotid surgery, or endovascular therapy) [26], while post-ischemic administration was designed to model an acute therapeutic window. Moreover, in vitro models using human induced pluripotent stem cell (iPSC)-derived brain microvascular endothelial cells (iBMECs), primary human astrocytes, and primary human pericytes enable us to evaluate the effects of metabolite exposure [27,28]. A BBB-on-a-chip model was used to investigate the influence of TUDCA on the barrier integrity and function. Furthermore, hypoxia was applied either before or after metabolite incubation to model distinct phases of ischemic stroke, allowing evaluation of TUDCA as both a prophylactic intervention before ischemic injury and a therapeutic intervention after the onset of ischemia. By combining the data from rodent tMCAO and dMCAO models with the human BBB-on-a-chip model, we provide here a more comprehensive framework for evaluating both primary and BBB-specific outcomes in the context of ischemia. Through this multi-model approach, we aimed to determine whether pre- and post-treatment with TUDCA provides neuroprotection and preserves BBB integrity following ischemic stroke and further ameliorates infarct size and behavioural outcomes.

## 2. Results

### 2.1. Administration of TUDCA Prior to Induction of tMCAO and dMCAO Does Not Alter Infarct Volume or Mitigate BBB Disruption

**Figure 1:**
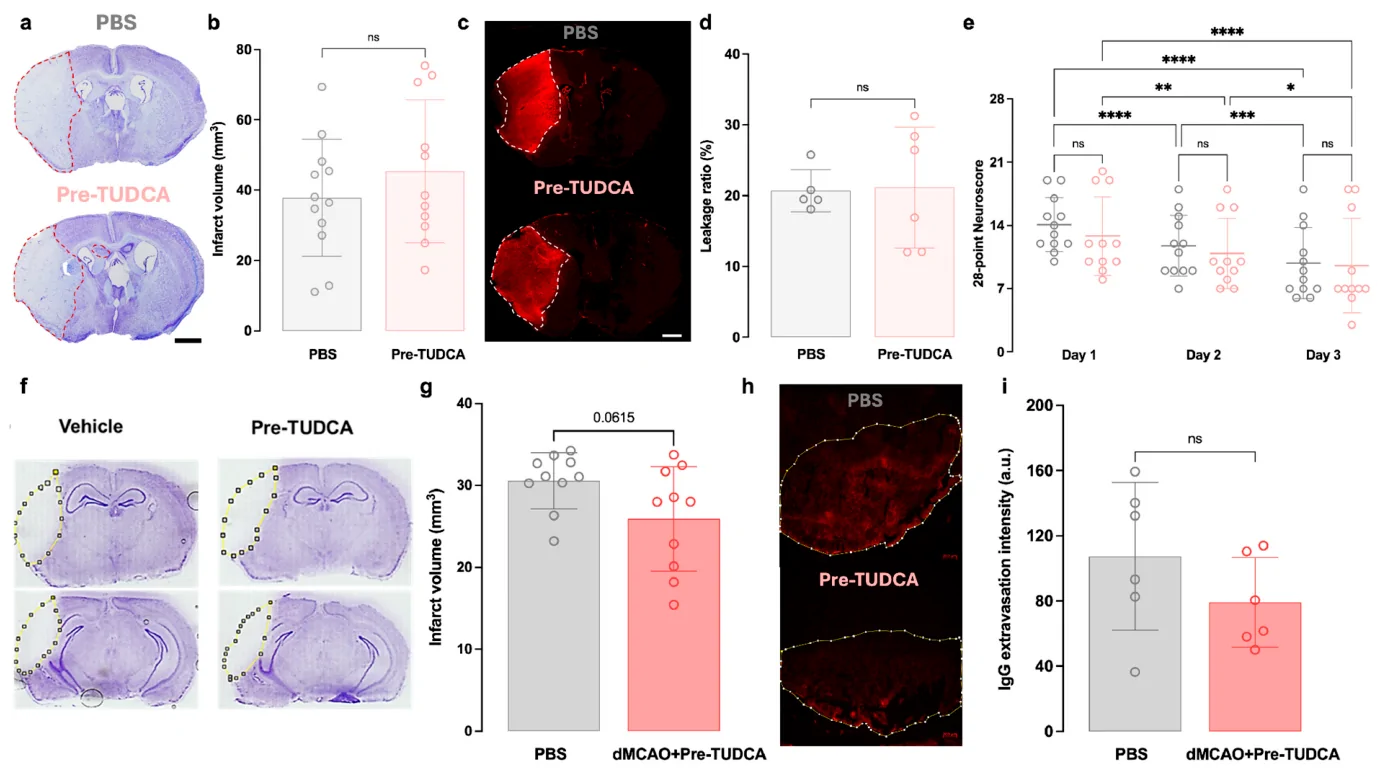
Assessment of cerebral infarction and neurological outcomes following TUDCA pretreatment in the tMCAO and dMCAO models. (**a**) Representative coronal brain sections stained with Cresyl violet from each experimental group subjected to the tMCAO surgery; dashed red lines show the stroke-induced lesion areas. Scale bar: 1 mm. (**b**) Infarct volumes were determined by measuring lesion areas across serial sections multiplied by the distance (PBS: *n* = 12; pre-TUDCA: *n* = 11). Statistical comparison was performed using an unpaired *t*-test. (**c**) Endogenous IgG immunofluorescence images representative of each group; dashed white lines indicate regions of IgG leakage. Scale bar: 1 mm. (**d**) BBB permeability was expressed as the proportion of IgG-positive area normalized to total brain area (PBS: *n* = 5; pre-TUDCA: *n* = 6); unpaired *t*-test. (**e**) Daily neurological assessments using the 28-point neuroscore, where 28 reflects maximum deficit and 0 denotes intact function (PBS: *n* = 12; pre-TUDCA: *n* = 11); two-way ANOVA with repeated measures. (**f**) Representation of cresyl violet staining of the brain section from both the PBS-treated and pre-TUDCA-treated animals subjected to dMCAO surgery; dashed yellow lines indicate the infarct area. (**g**) Infarct quantification showed no significant changes between the vehicle- and pre-TUDCA-treated animals (PBS: *n* = 10; pre-TUDCA: *n* = 10); unpaired *t*-test. (**h**) Representative images of IgG immunofluorescence staining of each group from the dMCAO experiment with yellow lines demonstrating IgG penetration area. Scale bar: 200 µm. (**i**) Quantification of endogenous IgG extravasation intensity (PBS: *n* = 6; pre-TUDCA: *n* = 5); unpaired *t*-test. Graphs display mean ± SD; each point represents one animal. ns, not significant. * *p* < 0.05, ** *p* < 0.01, *** *p* < 0.001, **** *p* < 0.0001.

To assess the potential neuroprotective effect of the bile acid TUDCA on stroke, a single intravenous injection of TUDCA was given one hour before inducing stroke using transient or permanent occlusion of the MCA, tMCAO and dMCAO, respectively, as a preventative strategy. Pre-treatment with TUDCA did not affect infarct volume in the ischemia/reperfusion tMCAO model (Figure 1a,b), an effect that was consistent across the rostro-caudal extent of the lesion and across individual brain regions (Supplementary Figure S1a,b). Assessment of neurological function was conducted using a standardized 28-point scoring system during the 3-day experiment, where 28 represents maximum deficiency, and 0 reflects the absence of apparent dysfunction. During the trial, the majority of animals exhibited spontaneous recovery; nevertheless, no statistically significant differences emerged between the pre-TUDCA-conditioned mice and the PBS group (Figure 1e). In contrast, in the permanent occlusion model (dMCAO), pre-treatment with TUDCA decreased the infarct volume, although this did not reach statistical significance (Figure 1f,g and Supplementary Figure S3).

To further examine stroke-induced BBB disruption in both in vivo models, the infiltration of endogenous large plasma proteins into the brain parenchyma (IgG extravasation) was evaluated using immunofluorescence labeling as a marker of vascular leakage. No substantial difference was observed between the groups in the transient mouse stroke model (Figure 1c,d). Nevertheless, TUDCA-treated mice subjected to permanent distal surgery exhibited a tendency for reduced IgG staining intensity compared to the vehicle group, yet this difference did not achieve statistical significance (Figure 1h,i). These findings indicate that TUDCA given before stroke does not influence stroke outcome and BBB leakage in mouse models of ischemia with and without reperfusion.

### 2.2. Administration of TUDCA After Stroke Does Not Significantly Diminish Infarct Volume or Attenuate Enhanced BBB Leakage

**Figure 2:**
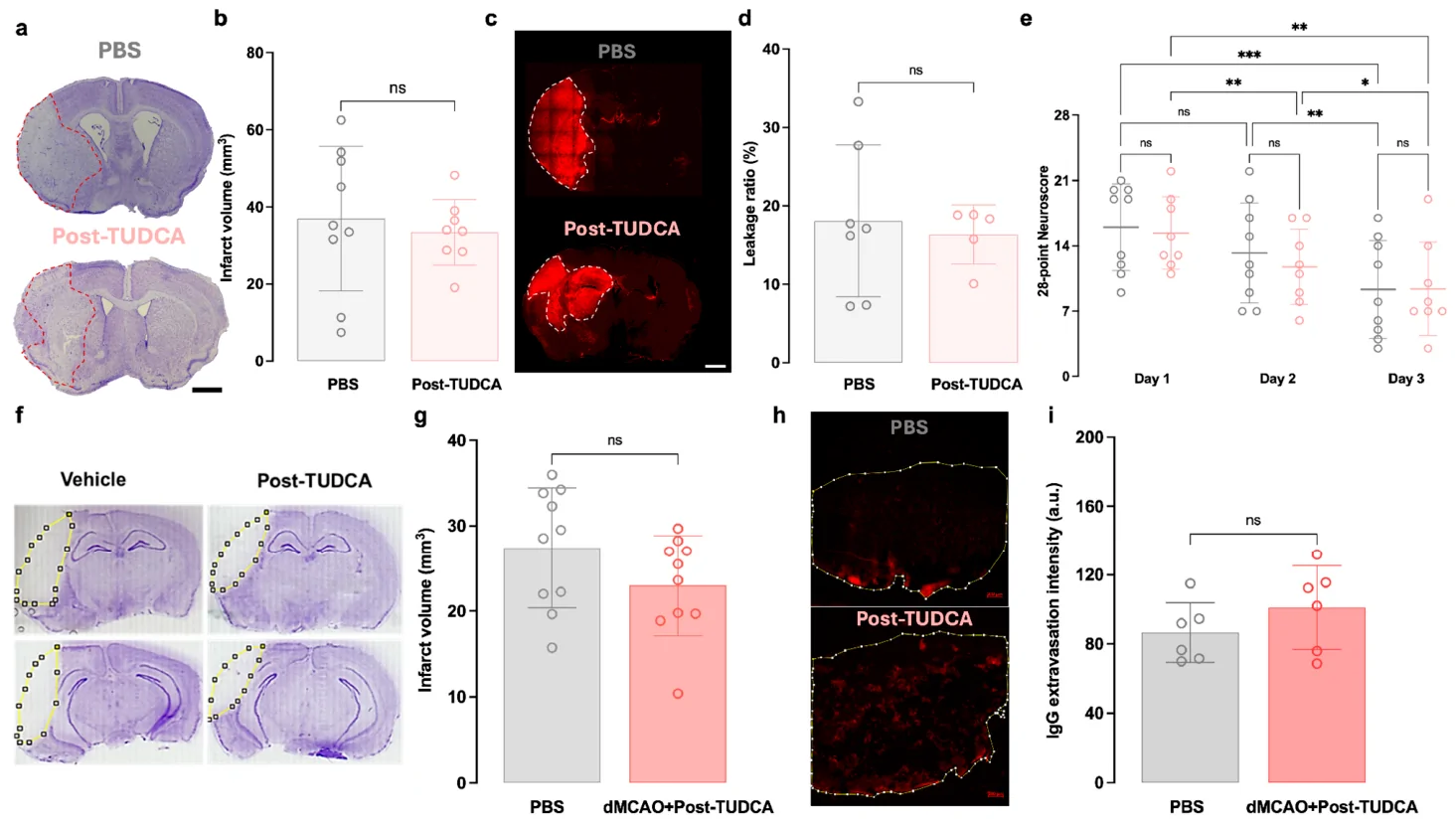
In vivo assessment of infarct extent, BBB integrity, and behavioural recovery in the TUDCA post-treatment experiment. (**a**) Coronal brain sections stained with cresyl violet from representative PBS- (top) and TUDCA-treated (bottom) animals administered post-MCAO; red dashed lines delineate the ischemic territory. Scale bar: 1 mm. (**b**) Total infarct ratio quantified in mice receiving either PBS (*n* = 9) or TUDCA (*n* = 8) after surgery. Unpaired *t*-test. (**c**) Immunofluorescence images representing endogenous IgG extravasation into the brain parenchyma in representative PBS- and TUDCA-injected animals post-surgery; white dashed lines mark the area of leakage. Scale bar: 1 mm. (**d**) IgG extravasation ratio determined for PBS- (*n* = 7) and TUDCA-injected animals (*n* = 5). Unpaired *t*-test. (**e**) Longitudinal assessment of neurological recovery post-MCAO using the 28-point neuroscore in PBS- (*n* = 9) and TUDCA-treated (*n* = 8) animals; two-way ANOVA with repeated measures. (**f**) Representation of cresyl violet staining of the brain sections from both the PBS- and post-TUDCA-treated groups obtained from the dMCAO experiment. Dashed yellow lines show the infarct area. (**g**) Infarct volume quantification showed no improvement between the vehicle (*n* = 10) and post-treatment (*n* = 10) groups; unpaired *t*-test. (**h**) Representative brain coronal sections stained with IgG; with yellow lines delineating the IgG-positive area. Scale bar: 200 µm. (**i**) Measurement of IgG extravasation intensity into the brain parenchyma compared between PBS and post-TUDCA-treated animals subjected to dMCAO (PBS: *n* = 6; post-TUDCA: *n* = 6). Statistical comparison was performed using an unpaired *t*-test. Data are expressed as mean ± SD. Each point represents one animal. ns, not significant. * *p* < 0.05, ** *p* < 0.01, *** *p* < 0.001.

To establish a more effective translational approach, we administered 100 mg/kg of TUDCA or an equivalent volume of PBS via the tail vein 1 h post-stroke induction in both in vivo models to assess the therapeutic efficacy of this secondary bile acid. Although there was a minor but consistent decrease in infarct volume after dMCAO, this was not statistically significant between TUDCA- and PBS-treated animals (Figure 2f,g and Supplementary Figure S3). In mice subjected to tMCAO that were post-treated with TUDCA, no differences were observed in infarct volumes (Figure 2a,b), with no regional or rostro-caudal differences detected between groups (Supplementary Figure S2a,b). Similarly, in accordance with the preventive experimental framework, neuroscoring was conducted daily following tMCAO surgery until the third day in the post-TUDCA paradigm. Although the majority of animals in both groups exhibited recovery compared to day 1 after surgery, no statistically significant differences were detected among groups at the time points tested post-stroke (Figure 2e). Furthermore, to assess the degree of plasma protein infiltration in the ipsilateral hemisphere, we quantified the IgG leakage ratio in brain samples obtained from the tMCAO procedure. Immunofluorescence labeling revealed no statistically significant differences between the groups (Figure 2d). Moreover, quantitative assessment of IgG staining intensity in the permanent ischemia model revealed a non-statistically significant increase in signal detection in the TUDCA-treated group (Figure 2i). Altogether, ischemia-induced neuronal death and neurological impairment were not improved following post-TUDCA treatment.

### 2.3. TUDCA Does Not Affect the Permeability of Human Cell-Based BBB-on-a-Chip

**Figure 3:**
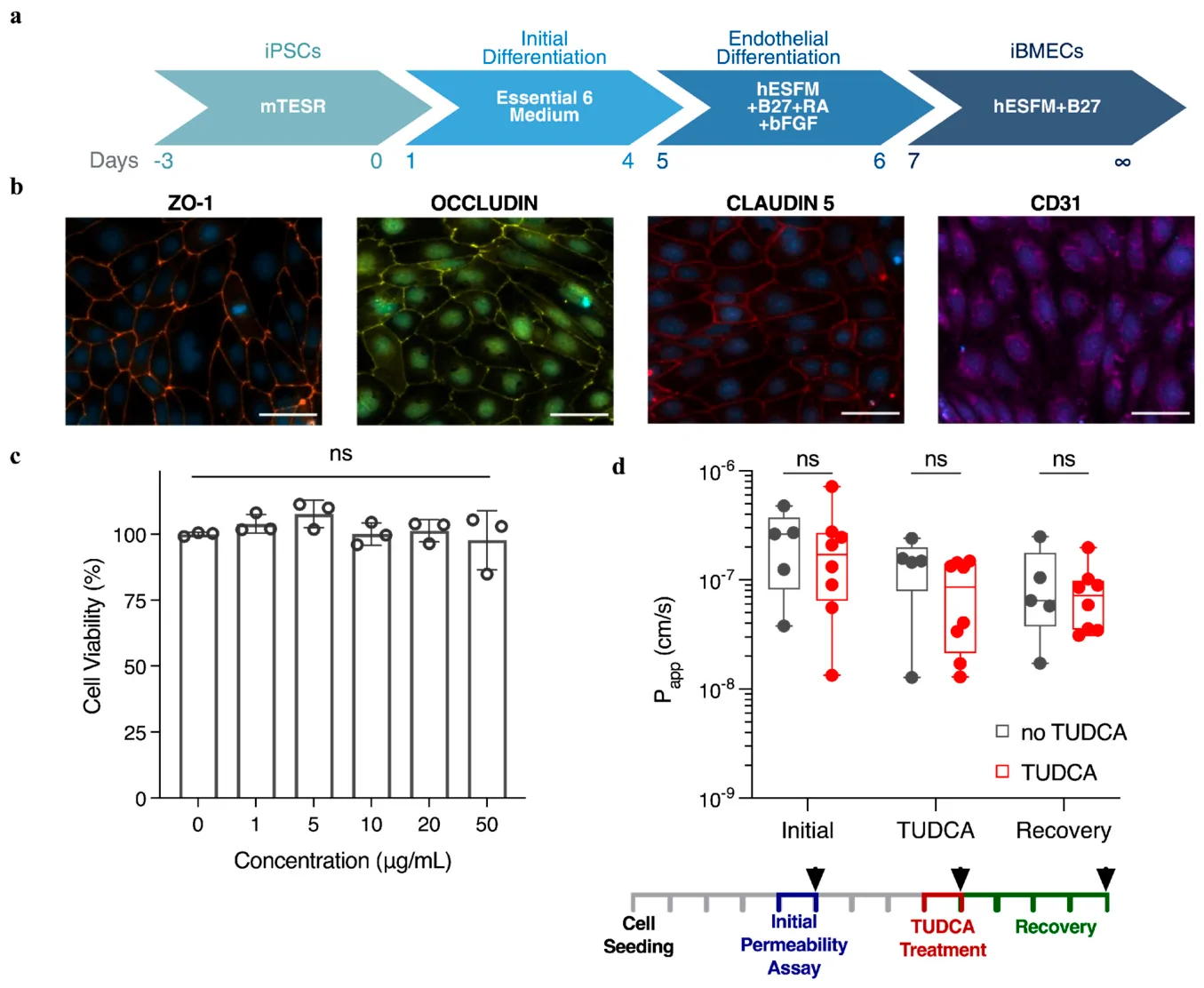
Differentiation of iBMECs and evaluation of TUDCA toxicity. (**a**) Schematic of the iBMEC differentiation protocol from human iPSCs. (**b**) Expression of tight junction proteins (ZO-1, occludin, and claudin-5) and endothelial marker CD31 in iBMECs. Nuclei of the cells stained with DAPI, shown in blue. Scale bar: 50 µm. (**c**) Cytotoxicity assessment of TUDCA on iBMECs using an MTT assay. *n* = 3 for each concentration. The statistical comparison was performed using one-way ANOVA. The plots are presented as mean ± SD. (**d**) Barrier integrity was evaluated by apparent permeability (P_app_) of 10 kDa FITC-dextran before, during, and after TUDCA treatment. Each dot represents a chip (*n* = 5 for no TUDCA, *n* = 7 for TUDCA). The experimental timeline is shown beneath the barrier integrity graph; each tick mark represents a 6-h interval, and arrows indicate the time points at which samples were collected for permeability measurements. Two-way ANOVA was performed for statistical analysis. The whiskers of the box plot show the minimum and maximum values. ns indicates no significant difference between groups.

Despite not observing a global effect on blood IgG extravasation to the brain parenchyma in mice treated with TUDCA, we asked whether more discrete functional and cellular features of the BBB were modulated by TUDCA using an in vitro human cell-based BBB model. To obtain human brain microvascular endothelial cells (BMECs) capable of forming a tight barrier, cells were differentiated from iPSCs following an established differentiation protocol, and the resulting cells were termed iBMECs (Figure 3a). The expression of tight junction proteins ZO-1, occludin, and claudin-5 in iBMECs confirmed successful differentiation (Figure 3b). The cytotoxicity of TUDCA on iBMECs was evaluated using an MTT assay, demonstrating that TUDCA is non-toxic at concentrations of 1, 5, 10, 20, and 50 µg/mL (Figure 3c). The slight fluctuations observed across doses were not statistically significant, likely reflecting minor inter-well variability.

To establish a complete human BBB model, human primary astrocytes and pericytes were co-cultured in the lower compartment of double-channel microfluidic chips, while iBMECs were seeded in the upper channel. BBB permeability was assessed using a permeability assay with a fluorescent tracer (10 kDa FITC-dextran) before, during, and after TUDCA treatment, followed by spectrophotometric analysis. These conditions mimic the pre-TUDCA treatment paradigm that was used in vivo. At the initial phase, well-established BBB chips were observed with apparent permeability (P_app_) values lower than 6 × 10^−7^ cm/s across all chips. Following 6 h of TUDCA treatment, the P_app_ values declined further, indicating a trend toward improved BBB integrity; however, no statistically significant differences were observed between untreated and TUDCA-treated chips. At 24 h post-treatment (“recovery” without TUDCA exposure), TUDCA-treated chips maintained the trend in enhancement of barrier integrity (Figure 3d); yet, the lack of statistical significance precludes any conclusion regarding a direct effect of TUDCA on improvement of BBB function.

### 2.4. TUDCA Does Not Affect ZO-1 Expression in iBMECs Under Hypoxia

**Figure 4:**
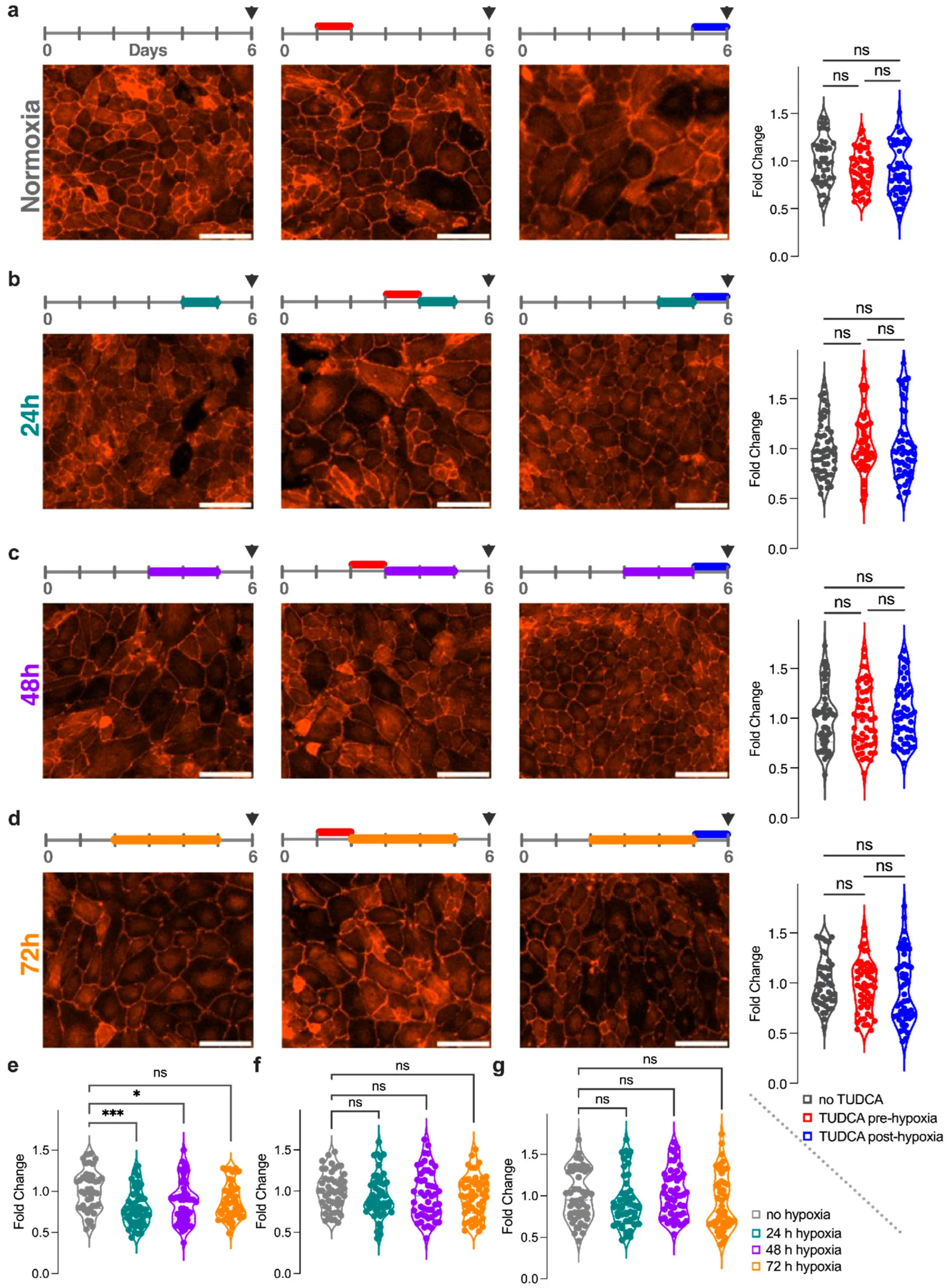
ZO-1 expression of iBMECs following TUDCA and hypoxia treatments. Hypoxic conditions were maintained in a hypoxic incubator with uninterrupted 5% O_2_ exposure for 24, 48 or 72 h. The experimental timeline is shown above each image, with each tick mark representing one day. Arrows indicate the imaging time point (Day 6). Red bars indicate TUDCA pre-treatment (20 µg/mL; before hypoxia), while blue bars indicate TUDCA post-treatment (20 µg/mL; after hypoxia). Green bars: 24 h hypoxia; purple bars: 48 h hypoxia; orange bars: 72 h hypoxia. Representative ZO-1 immunostaining images were shown for each condition. The y-axes shows fold change as mean fluorescence intensities normalized to “no TUDCA” or “no hypoxia” conditions. Rows correspond to (**a**) normoxia (no hypoxia), (**b**) 24 h hypoxia, (**c**) 48 h hypoxia, and (**d**) 72 h hypoxia, whereas columns correspond to untreated, TUDCA treatment before hypoxia, and TUDCA treatment after hypoxia. Quantification of normalized ZO-1 fluorescence intensity comparing the three treatment conditions within each row. (**e**) Comparison of normalized ZO-1 fluorescence intensity across oxygen conditions for untreated cells. (**f**) Comparison of normalized ZO-1 fluorescence intensity across oxygen conditions for cells treated with TUDCA before hypoxia. (**g**) Comparison of normalized ZO-1 fluorescence intensity across oxygen conditions for cells treated with TUDCA after hypoxia. Scale bar: 100 µm. Data were analyzed using ordinary one-way ANOVA and Tukey’s multiple comparisons test. ns, non-significant; * *p* < 0.0332; *** *p* < 0.0002.

To mimic a stroke-like microenvironment, iBMECs were exposed to hypoxic conditions (5% O_2_) for durations of 24, 48, or 72 h. TUDCA was administered either 24 h before hypoxia exposure (pre-treatment) or following hypoxia exposure (post-treatment). To correlate the iBMEC integrity alterations with the preceding permeability assessments, tight junction protein Zonula Occludens-1 (ZO-1) immunostaining was quantified on Day 6 as an indicator of the altered BBB integrity (Figure 4 and Supplementary Figure S4). ZO-1 was selected to complement the functional permeability measurements with an assessment of tight-junction integrity, rather than to comprehensively characterize ER-stress or apoptotic pathways. In the absence of TUDCA treatment, hypoxic exposure transiently decreased the fluorescence intensity of ZO-1 in a non-linear and time-dependent manner (Figure 4e). Notably, 24 h of hypoxic exposure induced the greatest reduction in tight junction integrity, whereas 48 h of hypoxia was associated with a lesser decrease in fluorescence intensity, and 72 h of hypoxia resulted in no considerable changes in ZO-1 expression. This pattern may suggest partial recovery of tight junction integrity beyond the initial 24-h hypoxic insult.

Despite observing a disruption in the tight junctions in the untreated hypoxic condition, pre-treatment with TUDCA did not result in ZO-1 expression changes between normoxic and hypoxic groups (Figure 4f). The same trend was observed in TUDCA post-treatment groups (Figure 4g). However, when group comparisons were performed within each hypoxia duration, no statistically significant differences were identified among the untreated, pre-treatment, and post-treatment conditions (Figure 4b–d). These results suggest that TUDCA does not significantly preserve the integrity of human-derived iBMECs under hypoxic conditions.

## 3. Discussion

The present study assessed the neuroprotective and BBB-preserving effects of microbial metabolites in the context of ischemic stroke, employing a multi-model approach using both in vivo and in vitro systems. For this purpose, tMCAO and dMCAO mouse models, together with a human BBB-on-a-chip system, were implemented to evaluate the effects of TUDCA, a secondary bile acid conjugated with taurine, on stroke outcome progression and BBB permeability. A major strength of this study lies in the use of randomized and blinded experimental designs, complemented by validation across independent experimental platforms. Such methodological rigor is widely advocated in the stroke research field to enhance reproducibility and bridge the translational gap between preclinical findings and clinical outcomes [29,30]. Contrary to our initial hypothesis and previous reports describing beneficial effects of TUDCA in the rat models of ischemic [8] and haemorrhagic stroke [9], data obtained from the current blinded and randomized study demonstrated that intravenous injection of TUDCA, administered 1 h before or after the onset of ischemia, did not significantly reduce infarct volume in an in vivo model of ischemia with and without reperfusion. Furthermore, no reduction in the extravasation of plasma proteins into brain parenchyma was observed in either model. Additionally, neurological deficit induced by tMCAO surgery was not ameliorated by pre-TUDCA treatment, nor by the post-treatment paradigm. In line with our in vivo findings, we could observe similar trends in the human BBB-on-a-chip system, where TUDCA treatment did not significantly alter barrier permeability under normoxic conditions, nor did it preserve ZO-1 expression in iBMECs exposed to hypoxia, regardless of its administration before or after hypoxic treatment. Importantly, the consistent observation of a lack of beneficial effects across multiple experimental platforms suggests that, under the conditions tested in this study, TUDCA has limited efficacy in preserving BBB function or improving stroke outcome.

While several studies have demonstrated beneficial effects of TUDCA supplementation in the context of neurological disorders by regulating ER stress, apoptosis, and mitochondrial dysfunction [10], we could not demonstrate the neuroprotective properties of exogenous administration of this secondary bile acid in our in vivo and in vitro models when injected before and after the induction of stroke or hypoxic exposure. Notably, the literature specifically examining TUDCA’s effects in models of cerebral stroke remains limited to the reports mentioned above, dating back over two decades. To our knowledge, no further studies have since reassessed TUDCA’s neuroprotective efficacy in the context of stroke, leaving few additional data points against which the present findings can be situated. Evidence from other disease contexts suggests that TUDCA’s efficacy is context dependent. A phase III clinical trial in amyotrophic lateral sclerosis (TUDCA-ALS, NCT03800524) was designed to assess whether TUDCA could slow disease progression [31]; findings indicate that TUDCA-treated patients did not show slower functional or motor decline compared to placebo, with no differences in survival time or biomarkers. Similarly, in a cholecystolithiasis study, the combination of TUDCA and intestinal probiotics did not improve the effects of either treatment alone, with intestinal probiotics showing superior efficacy in reversing bile composition and reducing gallstone formation [32]. Taken together, these findings indicate that the therapeutic potential of TUDCA may be restricted to pathological contexts and necessitates thorough optimization of dosage and administration strategies. Most importantly, the previously published observations were based on a rat MCAO model [8], whereas we employed mouse models of transient and permanent stroke. Such species-dependent variability has long been recognized as a major obstacle to the successful translation of preclinical neuroprotective strategies [33,34]. Evidence from the literature suggests that the neuroprotective effects of TUDCA may depend on species, dose, and treatment paradigm. In rats, acute TUDCA administration has been shown to exert neuroprotective effects following acute brain injury. For example, a single intravenous dose of TUDCA (300 mg/kg) administered 30 min after traumatic brain injury reduced brain lesion volume and cortical oedema [35]. Similarly, in experimental cerebral ischemia in rats, intravenous TUDCA administered shortly after ischemia onset was reported to reduce lesion size, with doses of 64–180 mg/kg producing approximately 30–40% lesion reduction and the highest tested dose of 400 mg/kg resulting in a reduction of approximately 50% [8]. In a rat model of hemorrhagic stroke, TUDCA doses ranging from 10 to 200 mg/kg were likewise reported to exert neuroprotective effects when administered before or after injury [9].

By contrast, studies demonstrating neuroprotective effects of TUDCA in mice have generally used prolonged treatment paradigms in neurological disease models distinct from acute cerebral ischemia. In APP/PS1 mice, intraperitoneal administration of TUDCA (500 mg/kg) every 3 days for 3 months initiated after disease onset, attenuated amyloid-β pathology [36], whereas daily oral administration of TUDCA (500 mg/kg) for 28 days ameliorated experimental autoimmune encephalomyelitis through a GPBAR1-dependent mechanism [37]. In the present mouse models of cerebral ischemia, however, acute administration of TUDCA 1 h before ischemia or during the post-ischemic period did not result in a significant neuroprotection.

The dose and treatment timing used in this study were selected based on previously reported efficacy of TUDCA in preclinical stroke models [8,9]. The selected dose fell within the range previously associated with neuroprotection in rats, while remaining below the highest dose tested in the ischemic stroke study [8]. Our post-ischemic treatment paradigm was designed to approximate the previously effective treatment window reported in the rat tMCAO study, in which TUDCA administered 1 or 2 h after ischemia onset exerted neuroprotective effects [8]. Pre-ischemic administration had not, to our knowledge, been examined in a comparable ischemic stroke model, although administration 1 h before injury was reported to be protective in a rat model of hemorrhagic stroke [9]. Despite using a dose and treatment windows informed by these previous studies, we did not reproduce the reported neuroprotective effects in our mouse models. Several factors may contribute to this discrepancy, including species-dependent sensitivity, dose responsiveness, treatment timing, and treatment duration. Importantly, because only a single TUDCA dose was evaluated in the present study, dose dependency cannot be determined, and the lack of neuroprotection does not exclude the possibility that higher or repeated doses might be required to achieve comparable biological effects in mice. Moreover, because species and treatment paradigms differ concurrently across the available studies, their individual contributions cannot be resolved from the existing literature. Direct comparisons using comparable dosing and treatment paradigms across species will therefore be required to determine the relative contribution of these factors.

The translational history of stroke therapeutics has repeatedly demonstrated that neuroprotective effects observed under specific experimental conditions are often difficult to reproduce across different study designs [29,38]. More broadly, seminal systematic analyses and more recent reviews have emphasized that neuroprotective efficacy in experimental stroke is strongly influenced by treatment timing and the relationship between drug administration, ischemia, and reperfusion [33,39]. Thus, although the current study incorporated clinically relevant treatment windows and a dose informed by previous stroke studies, alternative dose regimens or prolonged administration paradigms may be required to achieve sufficient biological activity within the ischemic brain. Furthermore, it should be noted that the present study was not designed to investigate the mechanistic pathways underlying the observed lack of therapeutic efficacy. Consequently, the molecular and cellular mechanisms responsible for the absence of an effect remain unclear. Accordingly, the therapeutic potential of TUDCA in ischemic stroke warrants further investigation through studies incorporating mechanistic analyses, alternative delivery routes, repeated administration strategies, and optimized dosing protocols.

## 4. Materials and Methods

### 4.1. In Vivo Experiments

Male C57BL/6J mice (aged 8–12 weeks) were purchased from Charles River, L’Arbresle, France, and maintained under standard laboratory conditions (e.g., temperature, humidity, and light/dark cycle) with ad libitum access to food and water. All experimental procedures related to the stroke experimental models in mice were performed in accordance with the Swiss Federal Veterinary Office and approved by the Vaud Cantonal Veterinary Office (license number VD2017.7d) and the competent governmental authority (Regierung von Oberbayern, Munich, Germany; Approval code: ROB-55.2-2532.Vet_02-20-167, Approval date: 15 March 2021; Approval code: TV 55.2-2532.Vet_02-21-138, Approval date: 7 November 2022). All animal experiments were carried out in compliance with the ARRIVE guidelines.

#### 4.1.1. Transient Middle Cerebral Artery Occlusion (tMCAO)

Animals were kept under anaesthesia using a face mask with a mixture of 70% N_2_O and 30% O_2_ containing 1.5–2% isoflurane. Their body temperature was maintained at 37.0 ± 0.5 °C using a heating pad and a rectal probe. Additionally, regional cerebral blood flow (rCBF) was continuously monitored throughout the procedure using laser Doppler flowmetry (Perimed AB, Järfälla, Sweden), with a probe fixed to the skull (located 1 mm posterior and 6 mm lateral to the bregma). Left MCAO surgery was performed using the intraluminal filament procedure, as previously described [40]. Briefly, a silicone-coated monofilament (Doccol Corp., Sharon, MA, USA) was inserted into the external carotid artery and advanced through the Internal carotid artery (ICA) until it occluded the origin of the MCA. The filament was held in place for 25 min and withdrawn after this period. To improve reperfusion, the common carotid artery ligation was removed after filament withdrawal. Successful surgery was defined when rCBF was below 20% of the initial level during the occlusion period and a reperfusion rate exceeding 50% of the baseline rate within 10 min of filament removal. The surviving mice were sacrificed with a lethal dose (150 mg/kg) of sodium pentobarbital at the end of the 72-h experiment. This was followed by intracardiac perfusion with 4% paraformaldehyde diluted in PBS at pH 7.4. The dissected brains were post-fixed overnight at 4 °C and cryoprotected in 30% sucrose for at least 48 h.

#### 4.1.2. Permanent Distal Middle Cerebral Artery Occlusion (dMCAO)

Permanent cortical ischemia was induced using the dMCAO model as described by Llovera et al. [41]. Briefly, following anesthesia, a 1 cm incision was made between the ear and the eye, and the temporal muscle was gently reflected to expose the temporal bone. The MCA was identified through the thinned temporal bone, which was carefully drilled and removed to visualize the vessel. The artery was coagulated in bipolar mode (7 W) proximal and distal to the bifurcation (or at two adjacent points if the bifurcation was not discernible). Vessel patency was reassessed after 30 s to exclude spontaneous recanalization, and coagulation was repeated if necessary. The temporal muscle was repositioned, the incision sutured, and animals were allowed to recover in a temperature-controlled environment (32 °C) before returning to their home cages. Postoperative analgesia (carprofen, 4 mg/kg) was administered daily from 24 h after surgery until day 4. On day 4 post-ischemia, mice were deeply anesthetized with ketamine (120 mg/kg) and xylazine (16 mg/kg) [42] and transcardially perfused with heparinized saline (2 U/mL). Brains were collected, snap-frozen on dry ice, and processed for infarct volumetry and subsequent histological analyses.

#### 4.1.3. Drug Administration

To evaluate the preventive properties of TUDCA (Selleck Chemicals LLC, Houston, TX, USA, Cat. No. S3654; Cayman Chemical Company, Ann Arbor, MI, USA, Item: 20277) in the tMCAO and dMCAO models, male C57BL/6J mice were randomized to receive a single intravenous (IV) injection of either TUDCA (100 mg/kg) or PBS one hour before ischemia induction by a blinded investigator. This dose was chosen because it falls within the range of previously tested doses (64–200 mg/kg) shown to confer neuroprotection in rat models of ischemic and haemorrhagic stroke [8,9], while remaining below the highest dose tested (400 mg/kg [8]) and corresponding to an injection volume suitable for tail-vein IV administration in mice. In the post-stroke TUDCA experiment, the same dose of TUDCA or PBS was administered intravenously one hour after reperfusion onset in the tMCAO model, and one hour after permanent occlusion onset in the dMCAO cohort, to assess the therapeutic effects of TUDCA. In the tMCAO model, this timing was deliberately chosen after completion of the ischemic occlusion period, to assess TUDCA in a post-reperfusion therapeutic setting.

#### 4.1.4. Infarct Volume Measurement

For the tMCAO study, 12 coronal cryostat sections (25 µm thick, spaced 700 µm apart) were obtained from each animal. Sections were stained with cresyl violet (CV) to visualize regions of neuronal damage and cerebral infarction [43]. Stained sections were imaged under a light stereomicroscope (Nikon SMZ25, Nikon Corporation, Tokyo, Japan) at 1.2× magnification and analyzed using Fiji software (version 1.54 f). Total infarct volume was calculated by summing the infarct areas across all sections and multiplying by the intersectional distance. To further characterize the extent and regional distribution of ischemic damage, infarct area was additionally analyzed across sequential coronal sections and infarct volume by brain region (cortex, striatum, hippocampus, thalamus, midbrain), for both the pre-treatment and post-treatment tMCAO paradigms (Supplementary Figures S1 and S2).

For the dMCAO cohort, brains were cryosectioned into 16–20 serial sections (20 µm thick, spaced 400 µm apart). CV-stained sections were digitized at a resolution of 600 dpi, and infarct volume was determined using the indirect method described by Swanson et al. (1990), in which edema-corrected infarct volume is calculated as the difference between the volume of the contralateral hemisphere and that of the intact ipsilateral tissue across serial sections [44]. Representative images of the distribution of the infarct lesion are shown in Supplementary Figure S3.

#### 4.1.5. Assessment of Blood–Brain Barrier Integrity

The permeability of the BBB was quantified using immunofluorescence labelling of extravasated IgG, as previously described [45]. In brief, several brain slices from the tMCAO paradigm were washed three times with PBS, blocked and permeabilised with blocking solution containing PBS,1% BSA, 0.1% Triton X-100, and 10% horse serum for 1 h at room temperature (RT). The slides were then incubated overnight at 4 °C with Alexa Fluor 594-conjugated goat anti-mouse IgG (Invitrogen, Waltham, MA, USA, Cat. No. A11032) in PBS containing 1% BSA, 0.1% Triton X-100, and 2% horse serum. Images were acquired using a Zeiss Z1 slide scanner (Carl Zeiss AG, Oberkochen, Germany) and analysed using QuPath software (version 0.5.1). The leakage ratio was calculated by summing the extravasated areas relative to the total brain areas and multiplying by 100.

The same staining protocol was applied to brain sections from the dMCAO cohort. Images, however, were acquired using a Zeiss Axio Observer Z1 fluorescence microscope (Carl Zeiss, Jena, Germany) with ZEN 2 software (ZEN 2.3 blue edition, Carl Zeiss Microscopy GmbH, Jena, Germany, 2011) and analyzed using Fiji software (version 1.54s). Vascular leakage was assessed by quantifying fluorescence staining within the ischemic core. Fluorescence signal was measured as corrected total cell fluorescence (CTCF) and expressed in arbitrary units (a.u.), representing the total background-corrected staining signal per region of interest (ischemic core). CTCF was calculated as follows: CTCF = Integrated Density − (Area × Mean Background Intensity).

#### 4.1.6. 28-Point Neuroscore

A standard test, the 28-point neuroscore, was used in the transient stroke model to monitor body symmetry, gait, climbing, spontaneous circling behaviour, forelimb symmetry, compulsory circling, and whisker response [46]. The test, ranging from 0 (healthy function) to 28 (maximum deficit), was conducted from day one until day three following MCAO.

### 4.2. In Vitro Experiments

#### 4.2.1. Maintaining Human iPSCs

Human-induced pluripotent stem cells (iPSCs, Stemcell, Panama City, Panama, SCTi003-A) were preserved in liquid nitrogen. Prior to thawing, a six-well plate was treated with 1:100 Cell Basement Membrane (CBM, ATCC, Manassas, VA, USA, ACS-3035) in DMEM/F-12 (Sigma, Capiz, Philippines, D8437-500 mL). The plate was thereafter incubated for one hour at 37 °C. iPSCs were thawed in a 37 °C water bath and subsequently diluted in DMEM/F-12. Cells underwent centrifugation at 2000× *g* for a duration of 3 min. The supernatant was removed, and the pellet was resuspended in mTeSR™1 pluripotent stem cell media (Stemcell, Panama City, Panama, 100-0276), supplemented with 10 µM ROCK inhibitor Y-27632 (Selleckchem, Houston, TX, USA, S1049). The CBM coating solution was removed before cell distribution onto the plates. The cells were incubated at 37 °C with a daily media replacement. Upon achieving confluency, the cells were detached utilising EZ-LiFT™ Stem Cell Passaging Reagent (Merck, Darmstadt, Germany, SCM139-500 mL). This was followed by an incubation period of 4 to 5 min at 37 °C. The samples were diluted with DMEM/F-12, subjected to centrifugation at 2000× *g* for 3 min, and then resuspended in mTeSR media before being seeded onto CBM-coated plates. The iPSCs were frozen by resuspending the pellet in NutriFreez^®^ D10 Cryopreservation Medium (Nutristem, Beit HaEmek, Israel, BI05-713-1E with the addition of 10 µM Y-27632. The cryovials were preserved in a Mr Frosty™ freezing container at −80 °C for 24 h and, thereafter, transferred to liquid nitrogen for long-term preservation.

#### 4.2.2. Differentiation of Human iPSCs into Human Brain Microvascular Endothelial Cells (iBMECs)

The 7-day differentiation protocol was initiated upon the attainment of confluency in iPSCs, ensuring that no overcrowding occurred. During the initial four days, the cells were cultured in Essential 6 (E6, Thermo Scientific, Waltham, MA, USA, A1516401) medium. On Days 5 and 6, the cells were transferred to Human Endothelial Serum-Free Medium (hESFM, Thermo Scientific, Waltham, MA, USA, 11111044) supplemented with 1X B27 (Thermo, A1895601), 10 µM all-trans retinoic acid (RA, Pitagen, Istanbul, Turkey, R2625-50mg), and 20 ng/mL basic fibroblast growth factor (bFGF, Merck, GF003). On Day 7, iBMECs were detached from the plate using Accutase Cell Detachment Solution (Merck, SCR005). Following a maximum 30-min incubation at 37 °C, the activity of Accutase was suppressed by DMEM/F-12, and cell counts were performed using 0.2% Trypan Blue (Sigma-Aldrich, MO, USA, T8154-100ML). The specified volume was centrifuged at 3000× *g* for 5 min and subsequently resuspended in hESFM+1X B27 (EC+B27). The residual iBMECs were cryopreserved in a freezing medium consisting of 60% heat-inactivated FBS (hi-FBS, Sigma, F7524; heat inactivation conducted at 60 °C for 30 min), 30% “EC+B27,” 10% DMSO (Serva, Heidelberg, Germany, 961.022.2500), and 10 µM Y-27632. The cells were cryopreserved in a Mr Frosty™ Freezing Container at −80 °C for one day before being promptly transferred to liquid nitrogen for a maximum duration of two weeks. To defrost iBMECs, the cells were submerged in a 37 °C water bath and diluted in DMEM/F-12. Following Trypan Blue counting, the requisite cell amount was centrifuged at 3000× *g* for 5 min, and the supernatant was resuspended in EC + B27. The residual iBMECs were cryopreserved once more as “passage 2 iBMECs,” although their viability diminished upon subsequent thawing.

#### 4.2.3. Assessment of BBB Integrity by Immunofluorescence

The 48-well plates were treated with 50 µg/mL Collagen-I (BUGAMED, BUGA-COL) and 100 µg/mL Fibronectin (Serva, Heidelberg, Germany, SE2137003) solubilised in DPBS containing calcium and magnesium (Serena, Cambridge, MA, USA, BDL-003). The plate was incubated at 37 °C overnight. 200,000 iBMECs were seeded in EC+B27. The experiment employed *n* = 2 wells for each condition. Prior to the treatment, the cells were cultured for 24–48 h, with a daily medium change. Upon completion of the experiment, each well of the 48-well plate was rinsed with 200 µL of DPBS with Ca and Mg. The cells were fixed using 100% ice-cold methanol (Sigma, 106009.25) for 20 min at room temperature, followed by two washes. Blocking was carried out with 1.5% bovine serum albumin (BSA, Pan Biotech, Aidenbach, Germany, p06-1403100) for one hour at RT. Primary antibodies for ZO-1 (zonula occludens-1, 1:1000, ProteinTech, Rosemont, IL, USA, 21773-1-AP), occludin (1:1000, ProteinTech, 27260-1-AP), claudin 5 (1:1000, ProteinTech, 29767-1), and CD31 (PECAM1, 1:750, ProteinTech, 11265-1-AP) were diluted in 1.5% BSA. The plate was incubated overnight at 4 °C. Subsequently, the wells were rinsed three times and then stained with Cy3 (1:1000, ProteinTech, SA00009-2) in 1.5% BSA for one hour at RT. The wells underwent three washings. Cell nuclei were stained with DAPI (1:1000, Sigma-Aldrich, MBD0015-5ML) diluted in DPBS with Ca and Mg at a 1:1000 ratio for 2 min at RT. A stock solution of TUDCA (Selleckchem, Houston, TX, USA) was prepared at a concentration of 3 mg/mL in DPBS with Ca^2+^ and Mg^2+^, with the pH calibrated to 7.25. Subsequently, 20 µg/mL of the stock solution was diluted in EC+B27. The plates containing iBMECs were positioned in a hypoxic incubator regulated at 37 °C with 5% CO_2_ and 5% O_2_ for uninterrupted hypoxic exposure for 24, 48 or 72 h. Although these time ranges do not represent ischemia durations in vivo, they were selected to investigate the BBB disruption under sustained hypoxic stress, and the potential effects of TUDCA under prolonged stress condition. Images were obtained using a Zeiss Axio Observer A1 at 10× magnification. Ten images were acquired for each condition, and five regions of interest (ROIs) were manually selected from each image, resulting in 50 fluorescence-intensity measurements per condition. Quantification was performed using Fiji software (version 1.54f) according to a previously described method [47], with minor modifications. Fluorescence intensity was measured directly from the raw fluorescence images within each ROI. The mean fluorescence intensity (MFI) was then calculated and normalized to the corresponding “no-hypoxia” or “no-TUDCA” controls. Group differences were assessed using either one-way or two-way ANOVA, as appropriate

#### 4.2.4. MTT Assay

A 96-well plate was coated with 50 µg/mL Collagen-I and 100 µg/mL Fibronectin in 50 µL of DPBS with Ca^2+^ and Mg^2+^ and incubated overnight. 50,000 iBMECs were seeded in EC+B27. Following 24 h, the medium was aspirated, and TUDCA was applied at doses of 0, 1, 5, 10, 20, and 50 µg/mL in EC+B27. After 24 h, the media were aspirated. 20 µL of a 5 mg/mL MTT (Merck, M2003) stock solution was diluted with 80 µL of EC+B27 in each well, achieving a final concentration of 1 mg/mL. Following a duration of 4 h, the MTT-containing fluids were aspirated, and 100 µL/well of DMSO was introduced. The plate was incubated on a shaker for 10 min. Absorbance values were quantified at 570 nm using a TECAN Infinite 200 Pro Plate Reader.

#### 4.2.5. Maintaining Astrocytes and Pericytes

Primary human astrocytes (ScienCell, Carlsbad, CA, USA, 1800) were cultured in T75 flasks using Astrocyte Medium enriched with 2% FBS, 1% Penicillin–Streptomycin, and 1% Astrocyte Growth Supplement (ScienCell, Carlsbad, CA, USA, 1801 AM). Primary brain vascular pericytes (ScienCell, Carlsbad, CA, USA, 1200) were cultivated in T75 flasks using Pericyte Medium supplemented with 2% FBS, 1% Penicillin–Streptomycin, and 1% Pericyte Growth Supplement (ScienCell, Carlsbad, CA, USA, 1201). The astrocytes and pericytes were used until passage 11. Cells were detached using Trypsin-EDTA (Pan Biotech, P10-024100/100 mL) solution at 90% confluency, followed by a 4–5-min incubation at 37 °C. Trypsin was suppressed using DMEM-F12 Medium or Astrocyte/Pericyte Medium, followed by centrifugation at 3000× *g* for 5 min. For routine maintenance, the supernatant was resuspended in Astrocyte/Pericyte medium and seeded in a flask/Petri dish for cell culture. The cells were cryopreserved in a freezing medium composed of Astrocyte/Pericyte Medium, DMSO, and heat-inactivated FBS in an 8:1:1 ratio.

#### 4.2.6. Design, Fabrication, and Culturing of the BBB Chips

Double-channel microfluidic chips were designed using SOLIDWORKS CAD. The microchannels were designed to be on top of each other, and each channel was 1.5 cm long, 1 mm wide, and 1 mm high. The molds were 3D-printed in Sabanci University Collaboration Space (CoSpace). SYLGARD 184 silicone elastomer kit (DS02 004008) was used to cast the chips. Briefly, the base was mixed with the curing agent at a 10:1 ratio. After 10–15 min of mixing, the mixture was exposed to vacuum to eliminate all air bubbles. The mixture was then poured into the molds, followed by another round of vacuum. The filled molds were incubated at 70 °C for at least 3 h.

The upper and lower channels were separated by a Sterlitech Corporation transparent polyester (PETE) membrane (Auburn, Washington, DC, USA) with 12 μm thickness and 0.4 μm pore size with 2 × 106 pore cm^2^ (PCT0420030). A 3-aminopropyltriethoxysilane-based method was used for the bonding [48]. Briefly, the PETE membranes were cut to the channel size. Then, the membranes were activated with PiezoBrush PZ3 O_2_ plasma for 30 s on each side, followed by immersion in 5% (3-Aminopropyl) triethoxysilane (APTES) solution (Sigma-Aldrich, 440140), diluted in DPBS +/+, for 20 min at 70 °C. Before PDMS-PETE bonding, the upper and lower PDMS compartments were washed with isopropanol (IPA) and dried with nitrogen gas. The PETE membranes were placed on filter papers to dry. Immediately before bonding, one side of the PETE membrane and one of the PDMS compartments were exposed to O_2_ plasma for 30 s. Then, the other PDMS compartment and the membrane-bound PDMS were treated with O_2_ plasma for 1 min. After that, the two compartments were immediately aligned using a microscope. Each bonded device was compressed and incubated at 100 °C for at least 12 h to complete the bonding protocol.

The chips were activated with direct UV exposure (365 nm) for a duration of 1 to 1.5 h, followed by coating with 50 µg/mL collagen-I and 100 µg/mL fibronectin in DPBS with Ca and Mg. The chips were incubated for 24 h at 37 °C. In the lower (“brain”) channel, 10,000/µL astrocytes were combined with 4286/µL pericytes in 10 µL of EC+B27, resulting in an astrocyte-to-pericyte ratio of 7:3. DPBS with Ca and Mg was retained in the top channel. The chips were inverted and incubated at 37 °C for 3 to 4 h. After 3–4 h, the chips were repositioned, and 200 µL micropipette tips were placed into one side of the top channel. iBMECs (10,000 cells/µL) in 30 µL of EC+B27 were introduced from the other end of the upper channel (“blood channel”) using a 200 µL pipette tip. The bottom channel was administered 30–50 µL of EC+B27 using the identical technique. The chips were incubated for a minimum of one day prior to commencing any studies. In the permeability assay, 50 µg/mL of 10 kDa fluorescein isothiocyanate-tagged dextran (FITC-dextran, Sigma, FD10S-100MG) was administered via the blood channel. The outflows from both channels were gathered, and the absorbance levels were quantified using a SpectraMax Gemini XPS Microplate Reader. The concentrations were determined by constructing a standard curve. The apparent permeability (P_app_) of static chips was determined using the following formula:

$$P_{\mathrm{app}}=\frac{V_{r}\;\times \;C_{r}}{∆t\;\times \;A\;\times \;C_{d0}}$$

where V_r_ is the volume of receiving channel outflow, C_r_ is the concentration in the receiving channel, A is the area of the membrane, t is the time in seconds, and Cd_0_ is the initial tracer concentration in the dosing channel. For TUDCA treatments, the stock was diluted in EC+B27 and administered through the blood channel with FITC-dextran.

#### 4.2.7. Statistical Analysis

All data are expressed as mean ± SEM. Statistical analyses were carried out in GraphPad Prism (version 8.3.0). Two-group comparisons were made using an unpaired Student’s *t*-test for normally distributed data or the Mann–Whitney U test where normality was not met. Treatment and time effects were evaluated using two-way repeated-measures ANOVA, with Sidak’s post hoc correction applied for multiple comparisons. For comparisons involving more than two groups, one-way or two-way ANOVA was applied as appropriate. A *p*-value < 0.05 was considered statistically significant.

## Data Availability

The original contributions presented in this study are included in the article. Further inquiries can be directed to the corresponding authors.

## Supplementary Materials

The following supporting information can be downloaded at: https://www.mdpi.com/article/10.3390/ijms27198702/s1.

## Abbreviations

The following abbreviations are used in this manuscript:

TUDCA	Tauroursodeoxycholic acid
BBB	Blood–brain barrier
MCAO	Middle cerebral artery occlusion
tMCAO	Transient middle cerebral artery occlusion
dMCAO	Distal (permanent) middle cerebral artery occlusion
MCA	Middle cerebral artery
ICA	Internal carotid artery
rCBF	Regional cerebral blood flow
IgG	Immunoglobulin G
ER	Endoplasmic reticulum
MMP	Matrix metalloproteinase
ZO-1	Zonula occludens-1
CD31/PECAM1	Platelet endothelial cell adhesion molecule-1
CV	Cresyl violet
CTCF	Corrected total cell fluorescence
a.u.	Arbitrary units
RT	Room temperature
iPSC	Induced pluripotent stem cell
iBMEC	iPSC-derived brain microvascular endothelial cell
RA	All-trans retinoic acid
bFGF	Basic fibroblast growth factor
hESFM	Human endothelial serum-free medium
EC+B27	hESFM supplemented with 1X B27 (working endothelial cell medium)
CBM	Cell basement membrane (coating matrix)
ROCK	Rho-associated protein kinase (inhibitor: Y-27632)
hi-FBS	Heat-inactivated fetal bovine serum
DMSO	Dimethyl sulfoxide
DPBS	Dulbecco’s phosphate-buffered saline
BSA	Bovine serum albumin
DAPI	4′,6-diamidino-2-phenylindole (nuclear stain)
FI	Fluorescence intensity
ROI	Region of interest
MTT	3-(4,5-dimethylthiazol-2-yl)-2,5-diphenyltetrazolium bromide (viability assay)
PDMS	Polydimethylsiloxane
PETE	Polyester (membrane material)
APTES	(3-Aminopropyl) triethoxysilane
IPA	Isopropanol
FITC	Fluorescein isothiocyanate
P_app_	Apparent permeability
ANOVA	Analysis of variance
SEM	Standard error of the mean
IV	Intravenous
PBS	Phosphate-buffered saline
CO_2_/O_2_/N_2_O	Carbon dioxide/oxygen/nitrous oxide
