# Supplementary material for: Reassessment of Neuroprotective Efficacy of TUDCA in Ischemic Stroke: A Multi-Model In Vivo and In Vitro Study
Source: Int J Mol Sci. 2026 Sep 29;27(19):8702. doi: 10.3390/ijms27198702 (PMC13665468; doi:10.3390/ijms27198702)
Supplement: Supplementary file 1 [file ijms-27-08702-s001.zip › ijms-4565540-supplementary.pdf]

Article

Reassessment of Neuroprotective Efficacy of TUDCA in Ischemic Stroke: A Multi-Model In Vivo and In Vitro Study

Seyedeh Maryam Mousavi[1,2,†], Sumeyra Nuran Ozmen[3,†], Mujeeb Adekunle Adedokun[4,†], Gulin Baran[3], Ahmet Baki Sahin[3], Lorenz Hirt[1,2], Nur Mustafaoglu [3,5,6,*] and Corinne Benakis[4,*]

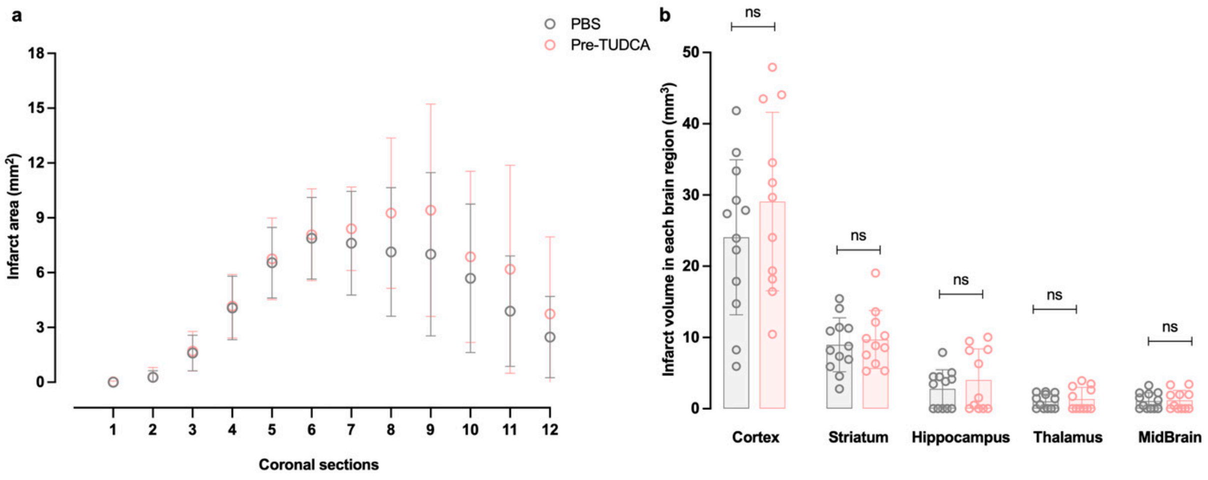

Supplementary Figure S1. Comprehensive cresyl violet staining analysis of infarct size after TUDCA pre-treatment in the tMCAO model. **(a)** Quantitative analysis of infarcted tissue across sequential coronal sections revealed the rostro-caudal profile of ischemic injury in TUDCA- and PBS-treated animals. Statistical significance was determined using two-way ANOVA with Sidak's multiple-comparisons test. **(b)** Infarct size was assessed regionally across different cerebral areas to characterize the regional distribution of ischemic injury in PBS- and TUDCA-pre-treated animals. Multiple unpaired *t*-tests were performed, with each data point representing an individual animal. (PBS: n = 12; pre-TUDCA: n = 11). Data are expressed as mean ± SD. ns, not significant.

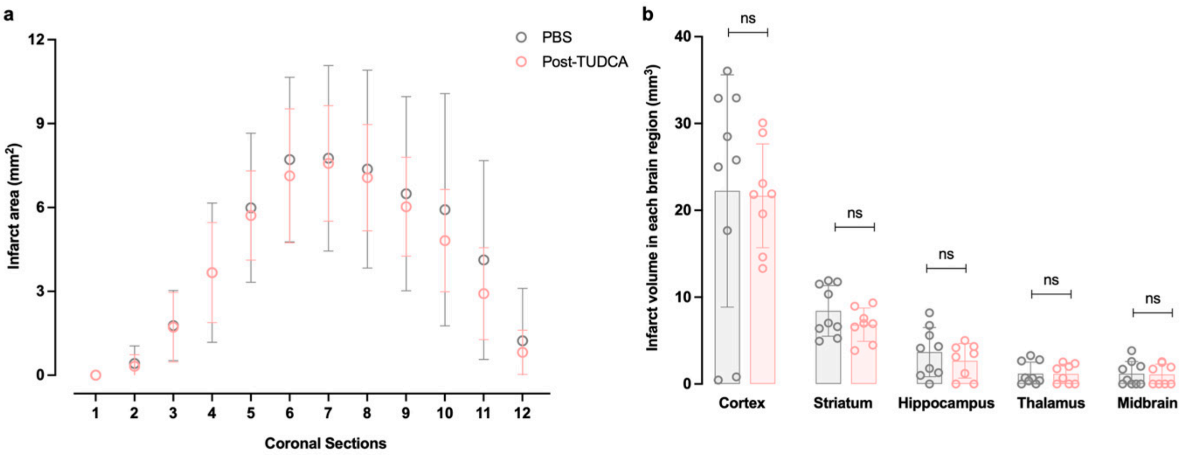

Supplementary Figure S2. Comprehensive assessment of infarct size following post-treatment with TUDCA in a preclinical model of transient stroke. (a) Infarct area was quantified across 12 coronal brain sections and compared between treatment groups using two-way ANOVA with Sidak's multiple-comparisons test. (b) Lesion volume was assessed across different brain regions to characterize the extent and distribution of ischemic injury following administration of TUDCA or PBS after ischemic onset. Multiple unpaired *t*-tests were performed, with each data point representing an individual animal. (PBS: $n = 9$; post-TUDCA: $n = 8$). Data are expressed as mean ± SD. ns, not significant.

.

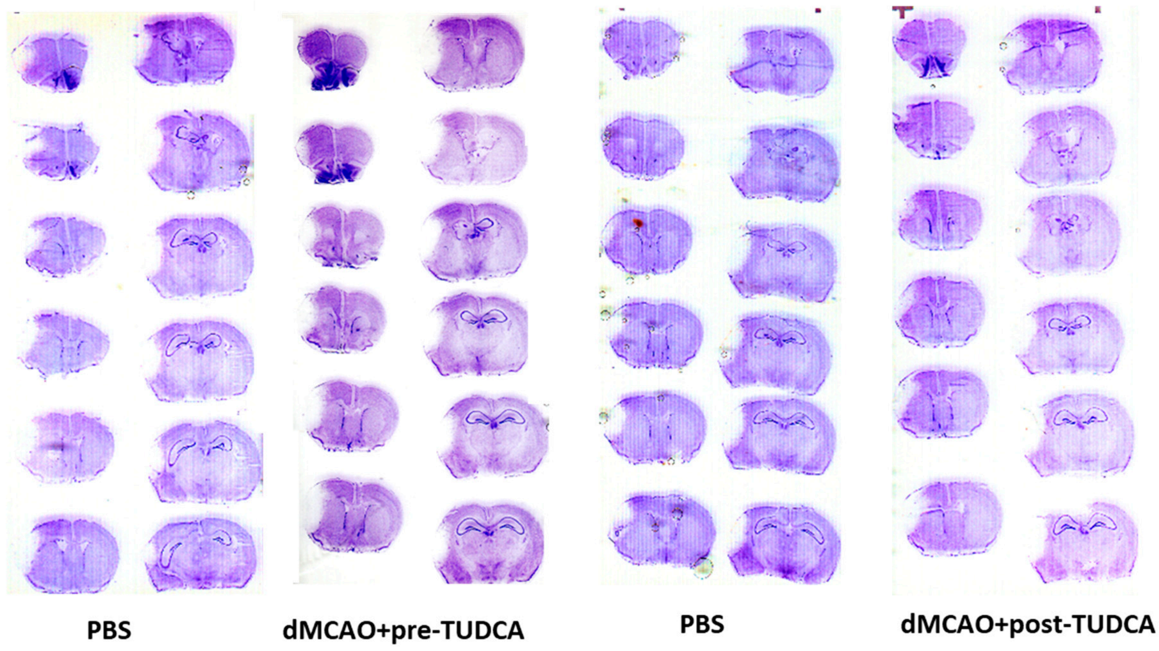

Supplementary Figure S3. Representative digitally scanned coronal brain sections following the dMCAO stroke model, showing cortical infarctions (white area) in each group based on Nissl-staining.

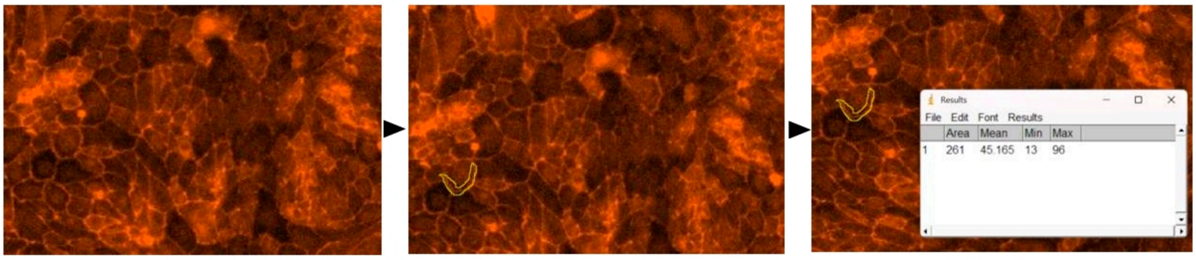

Supplementary Figure S4. Image analysis workflow used for quantitative analysis of Figure 4. Representative raw ZO-1 fluorescence image showing manual selection of a region of interest (ROI) and subsequent fluorescence-intensity measurement using Fiji. For each experimental condition, fluorescence intensity was measured in 50 manually selected ROIs on the raw fluorescence images. Mean fluorescence intensity (MFI) was calculated from these measurements and normalized to the corresponding no-hypoxia or no-TUDCA control.
